# *Aconiti Lateralis Radix Praeparata* as Potential Anticancer Herb: Bioactive Compounds and Molecular Mechanisms

**DOI:** 10.3389/fphar.2022.870282

**Published:** 2022-05-18

**Authors:** Wen Zhang, Chaoying Lu, Shuhui Cai, Yaru Feng, Jinjun Shan, Liuqing Di

**Affiliations:** ^1^ School of Pharmacy, Nanjing University of Chinese Medicine, Nanjing, China; ^2^ Jiangsu Engineering Research Center for Efficient Delivery System of TCM, Nanjing, China; ^3^ Jiangsu Key Laboratory of Pediatric Respiratory Disease, Institute of Pediatrics, Nanjing University of Chinese Medicine, Nanjing, China

**Keywords:** *Aconiti Lateralis Radix Praeparata*, Fuzi, alkaloid, polysaccharide, anticancer, mechanism

## Abstract

*Aconiti Lateralis Radix Praeparata* (Fuzi in Chinese) is a traditional herbal medicine widely used in China and other Asian countries. In clinical practice, it is often used to treat heart failure, rheumatoid arthritis, and different kinds of pains. Fuzi extract and its active ingredients exert considerable anticancer, anti-inflammatory, and analgesic effects. The main chemical substances of Fuzi include alkaloids, polysaccharides, flavonoids, fatty acids, and sterols. Among of them, alkaloids and polysaccharides are responsible for the anticancer efficacy. Most bioactive alkaloids in Fuzi possess C_19_ diterpenoid mother nucleus and these natural products show great potential for cancer therapy. Moreover, polysaccharides exert extraordinary tumor-suppressive functions. This review comprehensively summarized the active ingredients, antineoplastic effects, and molecular mechanisms of Fuzi by searching PubMed, Web of Science, ScienceDirect, and CNKI. The anticancer effects are largely attributed to inducing apoptosis and autophagy, inhibiting proliferation, migration and invasion, regulating body immunity, affecting energy metabolism, as well as reversing multidrug resistance. Meanwhile, several signaling pathways and biological processes are mainly involved, such as NF-κB, EMT, HIF-1, p38 MAPK, PI3K/AKT/mTOR, and TCA cycle. Collectively, alkaloids and polysaccharides in Fuzi might serve as attractive therapeutic candidates for the development of anticancer drugs. This review would lay a foundation and provide a basis for further basic research and clinical application of Fuzi.

## Introduction

With the incidence increasing year by year, malignant tumor has become one of the main factors that jeopardize human health. Currently, researchers are working on pathophysiology of cancer and seeking effective treatments, intending to continuously advance the progress of cancer therapy (Bray et al., 2018; Lee et al., 2018). Surgery, chemotherapy, and radiation are common methods to treat cancer. However, these treatments are often accompanied with adverse reactions and complications, such as fatigue, nausea, and pain. In addition, some commonly used chemotherapeutic drugs also have drug resistance, which hinders the treatment process (Gainor et al., 2016; Gainor et al., 2017). For the past few years, natural compounds extracted from plants have become more and more popular owing to their excellent anticancer effect and low toxicity. Many researchers are also committed to exploring natural compounds with anticancer activity as substitutes for chemotherapy (Guo et al., 2019; Rashid et al., 2019; Liu et al., 2021; Singh et al., 2021).

Fuzi is the processed product of the daughter root of *Aconitum carmichaeli* Debx, which has hot-natured and pungent in flavor recorded in *Shennong’s Classic of Materia Medica* firstly. Fuzi has been used as a traditional Chinese medicine (TCM) in China for centuries and offers therapeutic potential for heart failure, rheumatoid arthritis, gastroenteritis, depression, and other diseases (Wu et al., 2018; Meng-Qi Yang et al., 2019; Zhang et al., 2021). Constituents such as alkaloids, polysaccharides, flavonoids, fatty acids, ceramides, and trace elements are the material basis for Fuzi to exert a variety of functions (Wu et al., 2014). Studies indicated that alkaloids and polysaccharides in Fuzi possessed anticancer activity, which could availably induce tumor cell apoptosis, restrain cell proliferation, and regulate immunity. In addition, other compounds such as deltoin, sitosterol, neokadsuranic acid B, and 11,14-eicosadienoic acid were speculated to possess the potential to target PI3K/AKT pathway for anticancer effects under the prediction of network pharmacology and molecular docking (Xin Yang et al., 2019). In view of this, Fuzi alkaloids and polysaccharides have sparked increasing interest in the application of cancer therapy. A comprehensive perception of anticancer mechanisms is a prerequisite to the design of rational therapeutics. Therefore, the mechanisms account for the anticancer efficacies on Fuzi alkaloids and polysaccharides are required to be discussed and concluded. This review would provide a new recognition of Fuzi in treating cancer and be of great significance for guiding clinical medication and developing novel antineoplastic drugs.

## Fuzi Alkaloids

Pharmacological studies indicate that alkaloids in Fuzi have the effects of anti-inflammation, anticancer, immunoregulation, etc. (Li et al., 2019). The alkaloids in Fuzi are mainly diterpenoid ones, which are classified into three categories according to the number of carbon atoms on the mother nucleus: C_18_, C_19_, and C_20_ diterpenoid alkaloids (Zhu, 2008). Studies have demonstrated that diterpenoid alkaloids exert anticancer effects.

### C_18_ Diterpenoid Alkaloids

**FIGURE 1:**
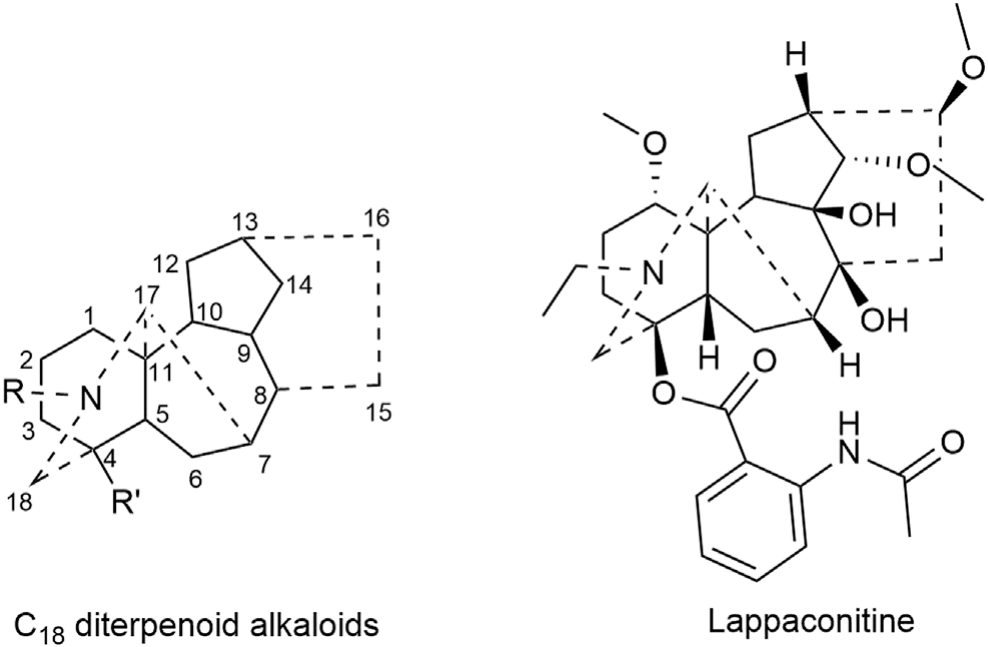
Structures of lappaconitine and C_18_ diterpenoid alkaloids in Fuzi.

Lappaconitine, a kind of C_18_ diterpenoid alkaloids, inhibited the proliferation of lung cancer A549 cells and induced cell apoptosis (Sheng et al., 2014; Fang, 2018). *In vivo* investigation revealed that the tumor inhibitory rate of mice bearing liver cancer is 11.20–53.08%; meanwhile that of mice bearing S180 sarcoma cells was 29.81–53.96% after the administration of lappaconitine (Lin et al., 2005). Besides, lappaconitine was widely used in the analgesia of various cancers with high safety and no addiction (Zhang et al., 2016; Sun, 2017). Figure 1 displays the structural formula of lappaconitine and the mother nucleus of C_18_ diterpenoid alkaloids in Fuzi.

### C_19_ Diterpenoid Alkaloids

**FIGURE 2:**
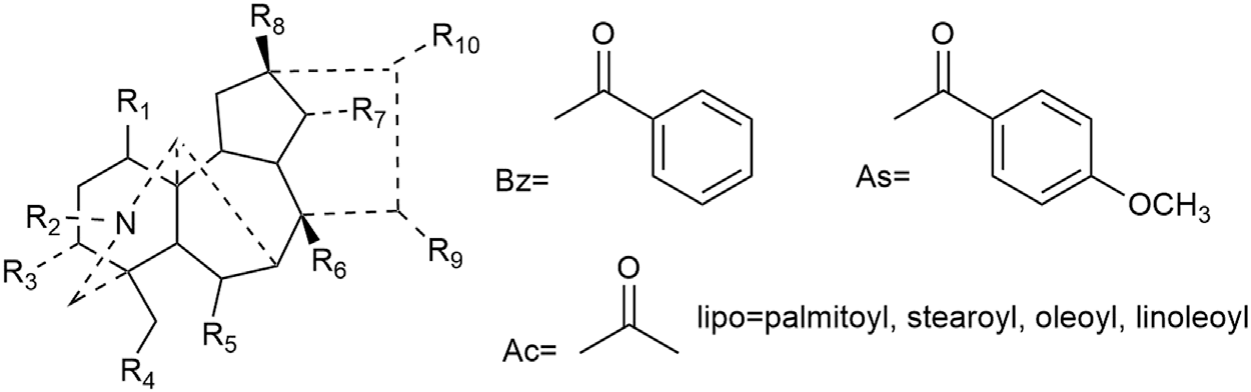
Structures of C_19_ diterpenoid alkaloids in Fuzi.

Most of the constituents with antineoplastic efficacies in Fuzi are C_19_ diterpenoid alkaloids. Apart from the bioactivity, these compounds are also poisonous, which lead to cardiotoxicity and neurotoxicity. Three main C_19_-diester-diterpenoid alkaloids (DDAs) were responsible for the toxicity of Fuzi, namely, aconitine, mesaconitine, and hypaconitine. Studies have shown that the median lethal dose (LD_50_) of single-dose oral aconitine in mice is 1.0–1.8 mg/kg, while the LD_50_ of mesaconitine and hypaconitine are 1.9 and 5.8 mg/kg, respectively. The dose–effect relationship of DDAs on clinical therapy remains to be further studied (Wada et al., 2005; Singhuber et al., 2009). So far, nearly 80 kinds of C_19_ diterpenoid alkaloids have been extracted (Tang et al., 2017; Zhang et al., 2020). The mother nucleus of C_19_ diterpenoid alkaloids is shown in Figure 2, and natural C_19_ diterpenoid alkaloids with anticancer activity are listed in Table 1. As shown in Table 1, the common ingredients of Fuzi, such as aconitine, mesaconitine, hypaconitine, benzoylaconitine, and neoline, have been proved to exhibit anticancer functions. Furthermore, the derivatives of C_19_ diterpenoid alkaloids could also repress the growth of tumor cells (Ren et al., 2017). The antiproliferative effects of C_19_ diterpene alkaloids and their derivatives on different tumor cell lines are summarized in Table 2. Among the tested cells, the HepG2 cells were highly sensitive to C_19_ diterpenoid alkaloids as well as their derivatives. By analyzing the structure–activity relationship, it was found that the alkaloids with stronger cytotoxicity, such as aconitine, mesaconitine, hypaconitine, crassicauline A, oxonitine, and dexyaconitine, were associated with the acetoxy group (OAc) on the substituent of R6, suggesting that the positions and species of substituents on the mother nucleus were closely related to the antitumor activity of C_19_ diterpenoid alkaloid.

**TABLE 1:** Structures of natural C_19_ diterpenoid alkaloids with anticancer activities in Fuzi.

No	Compounds	R_1_	R_2_	R_3_	R_4_	R_5_	R_6_	R_7_	R_8_	R_9_	R_10_	Reference
1	Aconitine	OMe	C_2_H_5_	OH	OMe	OMe	OA_C_	OB_Z_	OH	OH	OMe	Liu et al. (2004); Gao et al. (2012a); Qian (2015)
2	Crassicauline A	OMe	C_2_H_5_	H	OMe	OMe	OA_C_	OAs	OH	H	OMe	Gao et al. (2012a)
3	Deoxyaconitine	OMe	C_2_H_5_	H	OMe	OMe	OA_C_	OB_Z_	OH	OH	OMe	Gao et al. (2012a)
4	Hypaconitine	OMe	CH_3_	H	OMe	OMe	OA_C_	OB_Z_	OH	OH	OMe	Gao et al. (2012a)
5	Mesaconitine	OMe	CH_3_	OH	OMe	OMe	OA_C_	OB_Z_	OH	OH	OMe	Gao et al. (2012a)
6	Oxonitine	OMe	CHO	H	OMe	OMe	OA_C_	OB_Z_	OH	OH	OMe	Gao et al. (2012a)
7	Lipoaconitine	OMe	C_2_H_5_	OH	OMe	OMe	O-Lipo	OB_Z_	OH	OH	OMe	Wada and Yamashita, (2019)
8	Lipomesaconitine	OMe	CH_3_	OH	OMe	OMe	O-Lipo	OB_Z_	OH	OH	OMe	Wada and Yamashita, (2019)
9	Lipojesaconitine	OMe	C_2_H_5_	OH	OMe	OMe	O-Lipo	OAs	OH	OH	OMe	Wada and Yamashita, (2019)
10	Neoline	OH	C_2_H_5_	H	OMe	OMe	OH	OH	H	H	OMe	Hao (2014)
11	Benzoylaconitine	OMe	C_2_H_5_	OH	OMe	OMe	OH	OB_Z_	OH	OH	OMe	Shao et al. (2019)

**TABLE 2:** Cytotoxicity of C_19_ diterpenoid alkaloids and their derivatives in different cancer cell lines.

No	Compounds	Cell lines	Cancer types	IC_50_ (μM)	Reference
1	Aconitine	Hepal-6	Liver cancer	590.03	Qian (2015)
		KBv200	Drug-resistant KB subline	348.29	Liu et al. (2004)
		HCT8	Colon cancer	0.0812	Gao et al. (2012a)
		MCF7	Breast cancer	0.0245	
		HepG2	Liver cancer	0.0085	
2	Crassicauline A	HCT8	Colon cancer	0.1645	Gao et al. (2012a)
		MCF7	Breast cancer	0.1286	
		HepG2	Liver cancer	0.0236	
3	Oxonitine	HCT8	Colon cancer	0.2948	Gao et al. (2012a)
		MCF7	Breast cancer	0.0313	
		HepG2	Liver cancer	0.0861	
4	Deoxyaconitine	HCT8	Colon cancer	0.0514	Gao et al. (2012a)
		MCF7	Breast cancer	0.1035	
		HepG2	Liver cancer	0.0921	
5	Hypaconitine	HCT8	Colon cancer	0.1205	Gao et al. (2012a)
		MCF7	Breast cancer	0.0646	
		HepG2	Liver cancer	0.0092	
6	Mesaconitine	HCT8	Colon cancer	0.1316	Gao et al. (2012a)
		MCF7	Breast cancer	0.0457	
		HepG2	Liver cancer	0.0145	
7	Lipomesaconitine	A549	Lung cancer	17.2	Wada and Yamashita, (2019)
		MDA-MB-231	Breast cancer	20.0	
		MCF-7	Breast cancer	19.0	
		KB	Cervical carcinoma	10.0	
		KB-VIN	Vincristine-resistant KB subline	21.5	
8	Lipoaconitine	A549	Lung cancer	17.4	Wada and Yamashita, (2019)
		MDA-MB-231	Breast cancer	15.5	
		MCF-7	Breast cancer	16.0	
		KB	Cervical carcinoma	13.7	
		KB-VIN	Vincristine-resistant KB subline	20.3	
9	Lipojesaconitine	A549	Lung cancer	7.3	Wada and Yamashita, (2019)
		MDA-MB-231	Breast cancer	6.0	
		MCF-7	Breast cancer	6.7	
		KB	Cervical carcinoma	6.0	
		KB-VIN	Vincristine-resistant KB subline	18.6	
10	8-O-Azeloyl-14-benzoylaconine	HCT-15	Colon cancer	16.8	Chodoeva et al. (2005)
		A549	Lung cancer	19.4	
		MCF-7	Breast cancer	10.3	
11	Neoline	SGC-7901	Gastric cancer	37.55	Hao (2014)
		HepG2	Liver cancer	28.36	
		A549	Lung cancer	34.74	
12	14-O-Acetylneoline	SGC-7901	Gastric cancer	16.97	Hao (2014)
		HepG2	Liver cancer	33.76	
		A549	Lung cancer	18.75	

### C_20_ Diterpenoid Alkaloids

**FIGURE 3:**
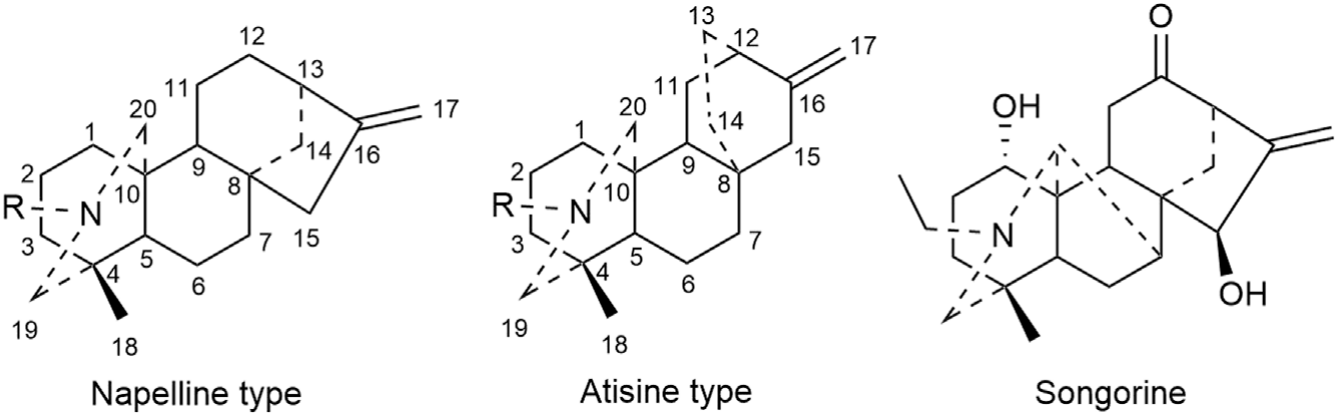
Structures of songorine and C_20_ diterpenoid alkaloids in Fuzi.

C_20_ diterpenoid alkaloid is another kind of alkaloid in Fuzi. Songorine, a natural C_20_ diterpenoid alkaloid found in Fuzi, has been proved to be antineoplastic. Studies revealed that songorine suppressed the proliferation of HepG2 cells effectively and increased apoptosis at both early and late stages (Sun et al., 2018). Moreover, songorine manifested other biological functions such as analgesic, anti-arrhythmic, and anti-inflammatory activities (Khan et al., 2018). Figure 3 shows the structural formula of songorine and two types of mother nucleus of C_20_ diterpenoid alkaloids in Fuzi. Based on the structure of natural C_20_ diterpenoid alkaloids, researchers synthesized a series of derivatives and tested their antiproliferative activity on different tumor cell lines. The results revealed that most derivatives exhibited considerable cytotoxic effects (Table 3). Malignant glioma A172 cells and lung cancer A549 cells were highly sensitive to most of C_20_ diterpenoid alkaloid derivatives. Of note, certain synthetic alkaloid derivatives displayed stronger anticancer activity than the natural ones. It was suggested that diterpenoid alkaloids demonstrated great potentiality in cancer treatment.

**TABLE 3:** Cytotoxicity of C_20_ diterpenoid alkaloids and their derivatives in different cancer cell lines.

No	Compounds	Cell lines	Cancer types	IC_50_ (μM)	Reference
1	Atisinium chloride	AGS	Gastric cancer	0.44	Zhu (2008)
		HepG2	Liver cancer	66.69	
		A549	Lung cancer	2.29	
2	15-Acetylsongoramine	HepG2	Liver cancer	0.59	Gao et al. (2012a)
3	Songorine	SGC-7901	Gastric cancer	46.55	Hao (2014)
		HepG2	Liver cancer	87.72	
		A549	Lung cancer	61.90	
4	12-Epi-napelline	SGC-7901	Gastric cancer	64.79	Hao (2014)
		HepG2	Liver cancer	96.99	
		A549	Lung cancer	65.91	
5	12-Epi-dehydronapelline	SGC-7901	Gastric cancer	65.00	Hao (2014)
		HepG2	Liver cancer	46.63	
		A549	Lung cancer	76.50	
6	12-Acetylluciculine	A172	Malignant glioma	13.95	Wada et al. (2007)
7	6,11-Dibenzoylpseudokobusine	A172	Malignant glioma	2.42	Wada et al. (2007)
8	11-Veratroylpseudokobusine	A172	Malignant glioma	2.52	Wada et al. (2007)
		A549	Lung cancer	3.5	Hazawa et al. (2009)
		A549	Lung cancer	4.07	Wada et al. (2011)
9	11-Cinnamoylpseudokobusine	A172	Malignant glioma	1.94	Wada et al. (2007)
		A549	Lung cancer	5.1	Hazawa et al. (2009)
		A549	Lung cancer	8.4 (GI_50_)	Wada et al. (2015)
		DU145	Prostate cancer	6.5 (GI_50_)	
		KB	Nasopharyngeal carcinoma	7.0 (GI_50_)	
		KB-VIN	Vincristine-resistant KB subline	6.4 (GI_50_)	
10	11-Anisoylpseudokobusine	A172	Malignant glioma	2.80	Wada et al. (2007)
		A549	Lung cancer	1.7	Hazawa et al. (2009)
		A549	Lung cancer	2.20	Wada et al. (2011)
		Raji	Lymphoma	5.18	Hazawa et al. (2011)
11	11-p-Nitrobenzoylpseudokobusine	A172	Malignant glioma	3.13	Wada et al. (2007)
		A549	Lung cancer	3.5	Hazawa et al. (2009)
12	11-(m-Trifluoromethylbenzoyl)pseudokobusine	A549	Lung cancer	4.4	Hazawa et al. (2009)
		Raji	Lymphoma	4.39	Hazawa et al. (2011)
13	6,11-Dianisoylpseudokobusine	A549	Lung cancer	3.68	Wada et al. (2011)
14	11,15-Dianisoylpseudokobusine	A549	Lung cancer	1.72	Wada et al. (2011)
15	11-p-Nitrobenzoate	A549	Lung cancer	5.08	Wada et al. (2011)
16	11,15-Di-p-nitrobenzoate	A549	Lung cancer	2.66	Wada et al. (2011)
17	11-Cinnamate	A549	Lung cancer	4.24	Wada et al. (2011)
18	11-m-Trifluoromethylbenzoate	A549	Lung cancer	4.67	Wada et al. (2011)
19	11-Anisoylkobusine	A549	Lung cancer	11.42	Wada et al. (2011)
20	11-(p-Trifluoromethylbenzoyl)kobusine	A549	Lung cancer	5.44	Wada et al. (2011)
21	11-(m-Trifluoromethylbenzoyl)kobusine	A549	Lung cancer	3.75	Wada et al. (2011)
22	11,15-Di-p-nitrobenzoylkobusine	A549	Lung cancer	3.02	Wada et al. (2011)
23	11,15-Dibenzoylkobusine	A549	Lung cancer	8.4 (GI_50_)	Wada et al. (2015)
		DU145	Prostate cancer	9.3 (GI_50_)	
		KB	Nasopharyngeal carcinoma	6.0 (GI_50_)	
		KB-VIN	Vincristine-resistant KB subline	7.5 (GI_50_)	
24	11,15-Dianisoylkobusine	A549	Lung cancer	6.7 (GI_50_)	Wada et al. (2015)
		DU145	Prostate cancer	7.1 (GI_50_)	
		KB	Nasopharyngeal carcinoma	5.3 (GI_50_)	
		KB-VIN	Vincristine-resistant KB subline	5.2 (GI_50_)	
25	11,15-Di-(4-nitrobenzoyl)kobusine	A549	Lung cancer	6.9 (GI_50_)	Wada et al. (2015)
		DU145	Prostate cancer	7.0 (GI_50_)	
		KB	Nasopharyngeal carcinoma	5.3 (GI_50_)	
		KB-VIN	Vincristine-resistant KB subline	5.5 (GI_50_)	
26	11,15-Di-(4-fluorobenzoyl)kobusine	A549	Lung cancer	8.1 (GI_50_)	Wada et al. (2015)
		DU145	Prostate cancer	6.8 (GI_50_)	
		KB	Nasopharyngeal carcinoma	5.2 (GI_50_)	
		KB-VIN	Vincristine-resistant KB subline	7.1 (GI_50_)	
27	11,15-Di-(3-trifluoromethylcinnamoyl)kobusine	A549	Lung cancer	5.5 (GI_50_)	Wada et al. (2015)
		DU145	Prostate cancer	6.2 (GI_50_)	
		KB	Nasopharyngeal carcinoma	4.1 (GI_50_)	
		KB-VIN	Vincristine-resistant KB subline	3.1 (GI_50_)	
28	11,15-Dibenzoylpseudokobusine	A549	Lung cancer	8.8 (GI_50_)	Wada et al. (2015)
		DU145	Prostate cancer	7.6 (GI_50_)	
		KB	Nasopharyngeal carcinoma	5.2 (GI_50_)	
		KB-VIN	Vincristine-resistant KB subline	6.3 (GI_50_)	
29	11-(4-Nitrobenzoyl)pseudokobusine	A549	Lung cancer	5.8 (GI_50_)	Wada et al. (2015)
		DU145	Prostate cancer	7.2 (GI_50_)	
		KB	Nasopharyngeal carcinoma	6.4 (GI_50_)	
		KB-VIN	Vincristine-resistant KB subline	6.4 (GI_50_)	
30	11,15-Di-(3-nitrobenzoyl)pseudokobusine	A549	Lung cancer	5.0 (GI_50_)	Wada et al. (2015)
		DU145	Prostate cancer	5.2 (GI_50_)	
		KB	Nasopharyngeal carcinoma	5.6 (GI_50_)	
		KB-VIN	Vincristine-resistant KB subline	5.6 (GI_50_)	
31	11-(3-Trifluoromethylbenzoyl)pseudokobusine	A549	Lung cancer	6.8 (GI_50_)	Wada et al. (2015)
		DU145	Prostate cancer	7.7 (GI_50_)	
		KB	Nasopharyngeal carcinoma	8.9 (GI_50_)	
		KB-VIN	Vincristine-resistant KB subline	6.2 (GI_50_)	
32	11-Tritylpseudokobusine	A549	Lung cancer	6.4 (GI_50_)	Wada et al. (2015)
		DU145	Prostate cancer	6.0 (GI_50_)	
		KB	Nasopharyngeal carcinoma	6.6 (GI_50_)	
		KB-VIN	Vincristine-resistant KB subline	5.2 (GI_50_)	

### Non-Diterpenoid Alkaloids

**FIGURE 4:**
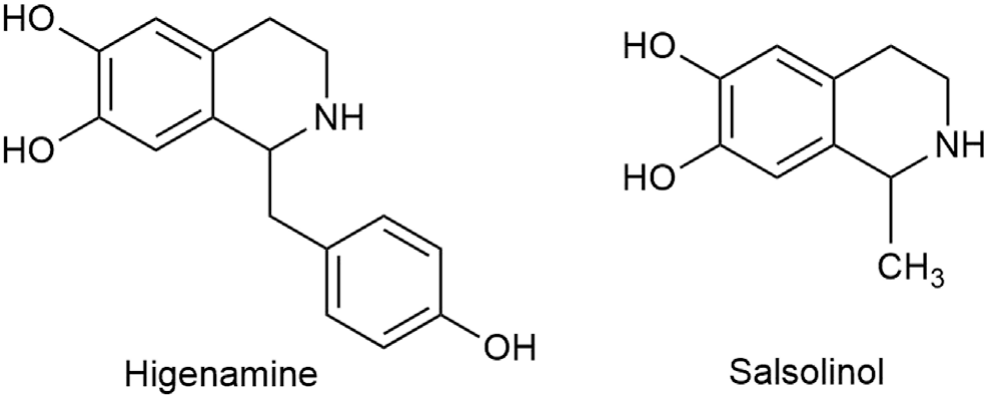
Structures of higenamine and salsolinol in Fuzi.

Apart from C_18_, C_19_, and C_20_ diterpenoid alkaloids, other types of alkaloids in Fuzi such as higenamine and salsolinol also manifested antitumor biological function. Higenamine enhanced the anticancer effects of cucurbitacin B in breast cancer by suppressing the interaction of protein kinase B (AKT) and cyclin-dependent kinase 2 (CDK2) (Jin et al., 2018). In colorectal cancer-bearing nude mice, higenamine inhibited tumor volume by inducing apoptosis (Song et al., 2020). Salsolinol, a water-soluble alkaloid in Fuzi, induced apoptosis of human neuroblastoma SH-SY5Y cells. After incubation with salsolinol, the levels of Bcl-2 decreased, while protein Bax and the release of cytochrome C increased, which led to mitochondrial dysfunction and apoptosis (Wanpen et al., 2007). Figure 4 displays the structures of higenamine and salsolinol.

## Fuzi Polysaccharides (FPS)

Polysaccharide is another key component of Fuzi, responsible for anti-inflammatory, anticancer, immunoregulatory, and cholesterol-lowering efficacies (Liu et al., 2019). FPS is mainly composed of glucose, galacturonic acid, galactose, arabinose, and mannose (Lu et al., 2011; Xu et al., 2014). One neutral FPS (FPS1) with 97% purity was isolated from Fuzi. FPS1 was composed of glucan as confirmed by acid hydrolysis, thin-layer chromatography, and infrared spectroscopy (Ruan et al., 2000). In addition, a water-soluble FPS (FPS2) was identified as a (1→3)-branched α-(1→6)-D-glucan. Through the study of structure–activity relationship, it was found that β-glucans and α-glucans consisting of (1→3)-glucopyranosyl units with (1→6)-linked side chains could activate leukocytes, stimulate the phagocytosis, and potentiate host response against many diseases such as cancer and infection. Likewise, in FPS2, α-glucans with similar linkage patterns displayed immunostimulatory activities as well (Zhao et al., 2006)**.**

So far, most studies considered that FPS exerted the antineoplastic effects indirectly by enhancing the immunity capacity. When FPS was used in combination with chemotherapeutic drugs, it exhibited synergism and increased potencies to promote the apoptosis of tumor cells (Dong et al., 2003a; Dong et al., 2003b; Gao et al., 2012b). FPS enhanced the anticancer activity of adriamycin long circulating liposome in treating liver cancer H22 mice by promoting lymphocyte proliferation and potentiating the cytotoxic index of natural killer (NK) cells (Dong et al., 2006). Furthermore, FPS prevented the proliferation and migration of tumor cells *via* restraining the synthesis of glycosyltransferase and matrix metalloproteinases (Gao et al., 2016; An et al., 2019).

## Anticancer Mechanisms of Fuzi Alkaloids and Polysaccharides

### Induce Apoptosis and Autophagy

As we know, apoptosis is crucial to the development of the body and the stability of internal environment. When carcinogenic driving factors lead to excessive cell proliferation, it usually triggers apoptosis (Green and Evan, 2002). Apoptosis is also called programmed cell death. The process of apoptosis is actually a cascade amplification process of irreversible hydrolysis of substrate by cysteinyl aspartate specific proteinase (caspase) (McIlwain et al., 2015), which could be divided into two major categories (intrinsic pathway and extrinsic pathway) (Carneiro and El-Deiry, 2020). As a lysosome degradation pathway with the function of nutritional circulation and metabolic adaptation, autophagy is considered to be involved in the regulation of cancer (Amaravadi et al., 2019). In many cases, autophagy is closely related to apoptosis, and autophagy-related genes are also involved in the process of apoptosis (Maheswari et al., 2018). Inducing apoptosis and autophagy is one of the primary mechanisms responsible for the anticancer effect of Fuzi alkaloids and polysaccharides.

#### Mitochondrial-Mediated Apoptosis

Mitochondria are the regulatory center of apoptosis. There is usually an increase in mitochondrial outer membrane permeability in the last step of apoptosis, accompanied by the release of cytochrome C (Cyto C) (Jin and El-Deiry, 2005; Kalkavan and Green, 2018). Mitochondrial-mediated apoptosis pathway (intrinsic pathway) is usually accompanied by changes in Bax, Bcl-2, Cyto C, ROS, caspase-3, etc. Bcl-2 and Bax are anti- and pro-apoptotic members of Bcl-2 family respectively, which could affect caspase cascade pathways in apoptosis. Cyto C is a key protein encoded by nuclear genes and its release is regulated by the members of Bcl-2 family (Li et al., 1997; Chao and Korsmeyer, 1998; Martinou and Youle, 2011). Aconitine promoted the release of Cyto C by directly increasing the production of reactive oxygen species (ROS) in mitochondria and ultimately induced cell apoptosis. This was demonstrated by the increased expression of caspase-3, caspase-7, Bax/Bcl-2, and PARP in HepG2 cells (Qi et al., 2018). Salsolinol (250 and 500 μM) caused the release of mitochondrial Cyto C in human neuroblastoma SH-SY5Y cells. At the same time, increased levels of Bax and decreased Bcl-2 were detected by western blotting analysis (Wanpen et al., 2007). Moreover, salsolinol enhanced the activity of caspase-3 in a time-dependent manner in SH-SY5Y cells (Jantas et al., 2008). NF-κB is a downstream gene of tumor necrosis factor α (TNF-α) pathway and participates in cell apoptosis (Covert et al., 2005). Studies demonstrated that a series of processes, such as inflammation, angiogenesis, invasion, and cellular metabolism, were controlled by the NF-κB pathway (Taniguchi and Karin, 2018). The activation of the pathway was observed in most kinds of cancers (Yu et al., 2020). The NF-κB pathway was involved in the apoptosis induced by Fuzi alkaloids. With the increment of aconitine, the expression of NF-κB and Bcl-2 decreased gradually, while the protein levels of Bax, caspase-3, caspase-9, PARP, and Cyto C increased (Ji et al., 2016). The research indicated that inhibiting the NF-κB pathway initiated the apoptosis program. Therefore, NF-κB might serve as a promising target in cancer therapy.

Endoplasmic reticulum (ER) dysfunction is to blame for ER-dominated apoptosis to a large extent. In cells, protein synthesis, processing, and modification depend heavily on ER. However, under the condition of viral infection and pH imbalance, the accumulation and aggregation of unfolded proteins would cause severe ER stress (ERS) (Hu et al., 2019). The apoptosis induced by C/EBP homologous protein (CHOP) pathway is a critical manner of ERS-mediated apoptosis, which is mainly regulated by kinase and transcription factors such as PKR-like endoplasmic reticulum kinase (PERK), eukaryotic translation initiation factor 2α (eIF2α), activating transcription factor 6 (ATF6), inositol-requiring enzyme-1 (IRE1), and X-box binding protein 1 (XBP1) (Oyadomari and Mori, 2004; Tabas and Ron, 2011; Sanchez-Lopez et al., 2013). The triggering of CHOP pathway is closely associated with mitochondria-mediated apoptosis, including increasing the expression of Bim and decreasing the expression of Bcl-2 (Hu et al., 2019). It was found that aconitine and quercetin increased apoptosis in human cervical cancer HeLa cells. The two components synergistically activated the glucose-regulated protein 78 (GRP78), a maker of ERS, eventually inducing ERS-mediated apoptosis by upregulating the PERK/eIF2α/ATF4/CHOP pathway (Li et al., 2018).

#### Death Receptor-Mediated Apoptosis

Death receptors on cell membranes play a crucial role in apoptosis. They induce apoptosis by binding to relevant ligands and transmitting apoptotic signals that eventually cause tumor cell death. Accordingly, the death receptor-mediated pathways could be applied to the field of tumor therapy (Yang, 2020). Death receptors are the initial part of extrinsic apoptosis, including Fas, TNF-R1/TNF-R2, and DR4/DR5, etc. (Locksley et al., 2001; MacEwan, 2002; Ashkenazi and Salvesen, 2014). Exogenous death receptor-mediated pathway initiates apoptosis by activating Fas, TNF-R, and other receptors on the cell membrane. When the death receptor binds to its ligand, the apoptosis signal can be transmitted to adaptor protein such as FADD, and then FADD binds to the initial protein caspase-8 or caspase-10, promoting the release of Cyto C and further triggering the cascade of caspase proteolysis (Lavrik et al., 2005; Billen et al., 2008; Walczak, 2013). Aconitine upregulated the expression of DR5 and TNF-R1 by targeting the p38 MAPK, accordingly activating Bax in A549 cells in a concentration-dependent manner, revealing that aconitine exerted the anticancer effects through the death receptor-mediated apoptosis (Fan et al., 2016). Studies showed that *p53* gene could induce apoptosis, regulate cell cycle, and repair DNA damage (Zhao et al., 2021). It is noteworthy that *p53* could directly participate in the transcription and regulation of death receptors, such as Fas and DR5 (El-Deiry, 1998). Mesaconitine induced apoptosis of leukemia daunorubicin-resistant cells, whose mechanism might be related to the upregulation of *p53* and caspase-3 (Guan et al., 2017). S180 sarcoma and H22 liver cancer mice experiments showed that both crude and acidic FPS inhibited neoplasm tissues growth significantly. Further research revealed that the two FPS increased tumor cell apoptosis by upregulating the expression of *p53* and Fas (Dong et al., 2003a).

#### Autophagy

Autophagy is a defense and stress-regulation mechanism of the body, which is essential for maintaining cell homeostasis. Autophagy would be triggered in states of excessive hunger, nutritional deficiency, oxidative stress, etc. (Amaravadi et al., 2016). Beclin-1 is a critical regulator of autophagy, which mainly participates in the formation of autophagosomes and could be blocked by Bcl-2 (Mariño et al., 2014). Studies indicated that oncogenic proteins inhibited the occurrence of autophagy. On the contrary, oncosuppressor proteins could activate autophagy (Galluzzi et al., 2015). The process of autophagy was initiated by the phosphorylated *Beclin-1* gene, and then gene *LC3* would be transformed into the membrane type *LC3-II*, eventually substrate p62 protein would be degraded (Onorati et al., 2018; Levine and Kroemer, 2019). It was reported that benzoylaconitine induced autophagy and apoptosis of human lung cancer A549 cells by upregulating Beclin-1, LC3-Ⅱ, Bax, and caspase-3, meanwhile downregulating p62 and Bcl-2 (Shao et al., 2019). The results suggested that Fuzi alkaloids could promote the expression of autophagy and pro-apoptotic-related proteins, also inhibit the anti-apoptotic protein such as Bcl-2 to conduct autophagy and apoptosis.

### Inhibit Proliferation, Migration, and Invasion

Proliferation, migration, and invasion are basic biological characteristics of malignancies. Uncontrolled proliferation, rapid migration, and excessive invasion of tumor cells are leading causes for poor prognosis and high recurrence. Accordingly, inhibition of these pathological processes would contribute to the anticancer efficacies of Fuzi. Studies have demonstrated that several processes and targets were involved in these pathological processes, including p38 MAPK, AKT, EMT, β3GnT8, and MMPs.

#### p38 MAPK

Mitogen-activated protein kinase (MAPK), composed of three kinase members, p38 MAPK, ERK, and JNK, could be activated by a variety of mitogens and sequentially induced cells enter into the division cycle. Researches revealed that p38 MAPK pathway took part in the regulation of cell proliferation, migration, and apoptosis (Martínez-Limón et al., 2020). MAPKAPK, a downstream target of p38 MAPK, directly induced phosphorylation of heat shock protein 27 (HSP27) and regulated cell migration, proliferation and apoptosis. Evidence was provided that p38 MAPK played a pivotal part in the occurrence and progression of cancer. Aconitine could significantly reduce the phosphorylation of p38, MAPKAPK, and HSP27 in liver cancer cells, indicating that aconitine suppressed the proliferation of MHCC97 cells by restraining the activation of p38 MAPK pathway (Xiong et al., 2018).

#### AKT

AKT, a serine/threonine kinase, is involved in cell growth, proliferation, apoptosis, survival, and glycogen metabolism (Datta et al., 1999). As an oncogenic protein, the activation of AKT is a general molecular biological feature in cancer (Hennessy et al., 2005; Shaw and Cantley, 2006; Song et al., 2019). Studies found that higenamine combined with cucurbitacin B blocked breast cancer cells in the G_2_/M phase. Furthermore, combination of the two ingredients decreased the expression of AKT and cell-cycle-related protein CDK2, indicating that higenamine possibly suppressed the proliferation of tumor cells by regulating the AKT signaling pathway negatively (Jin et al., 2018).

#### EMT

Epithelial–mesenchymal transition (EMT) refers to a biological process of epithelial cells transforming into cells with mesenchymal phenotype by specific procedures, which is linked to tumor invasion and migration. EMT is related to many mediators, such as transforming growth factor-β1 (TGF-β1), snail homolog 1 (Snail), and NF-κB pathways (Feng et al., 2020). Hypaconitine could significantly suppress TGF-β1-induced EMT in lung cancer A549 cells. Further investigation verified that hypaconitine attenuated nuclear translocation of NF-κB and reduced the expression of Snail, which showed a similar inhibitory effect on the NF-κB inhibitor. In the meantime, a significant decrease was observed in cell adhesion, invasion, and migration (Feng et al., 2017). The results suggested that hypoaconitine showed its anticancer potential via suppressing EMT.

#### β3GnT8

Studies observed that the aberrant glycosylation on the surface of tumor cells is a common feature of tumor malignant transformation and metastasis, which usually involves the modifications of terminal sialylation, fucosylation, O-glycan truncation, etc. (Bresalier et al., 1998; Pinho and Reis, 2015; Cagnoni et al., 2016; Girotti et al., 2020). Deregulation of glycosyltransferase in tumor cells leads to an altered glycan pattern of numerous proteins, resulting in abnormal glycosylation of proteins, and ultimately leading to the dysfunction of proteins, which might be involved in the metastasis of tumor cells (Oliveira-Ferrer et al., 2017). β1, 3-N-acetylglucosminyltransferase 8 (β3GnT8) is a glycosyltransferase that could catalyze the synthesis of polylactosamine chains and promote cell glycosylation. The polylactosamine chains and their related structures participated in the invasion and migration of tumor cells (Liu, 2017). It was found that FPS directly targeted the β3GnT8. The malignancy of tumor-bearing mice decreased along with a reduced expression of β3GnT8 and polylactosamine in liver cancer SK-HEP-1 cells after treatment with FPS, indicating that FPS inhibited the migration and invasion through downregulating β3GnT8 (Gao et al., 2016).

#### MMPs

Members of matrix metalloproteinases (MMPs) family mainly catalyze the proteolytic activities and thereby aid the breakdown of extracellular matrix (ECM), which is a vital tissue barrier for tumor metastasis (Vandenbroucke and Libert, 2014). MMPs are upregulated through all stages of cancer that could degrade various proteins in ECM and destroy the histological barrier against cell invasion (Isaacson et al., 2017). To date, 23 MMP family members have been found in humans. Numerous studies focus on the design of targeted antineoplastic agents by inhibiting MMPs (Cathcart et al., 2015; Das et al., 2021). MMP-2 and MMP-14, two members in the MMPs family, could degrade ECM, promote vascular proliferation, enhance cell migration and invasion, and are considered potential biomarkers of certain cancers (Alaseem et al., 2019; Karamanou et al., 2020). FPS reduced the tumor weight and blocked the expression of MMP-2 and MMP-14 in mice transplanted with gastric cancer, suggesting that FPS exerted anticancer effect by downregulating MMP-2 and MMP-14 (An et al., 2019).

**FIGURE 5:**
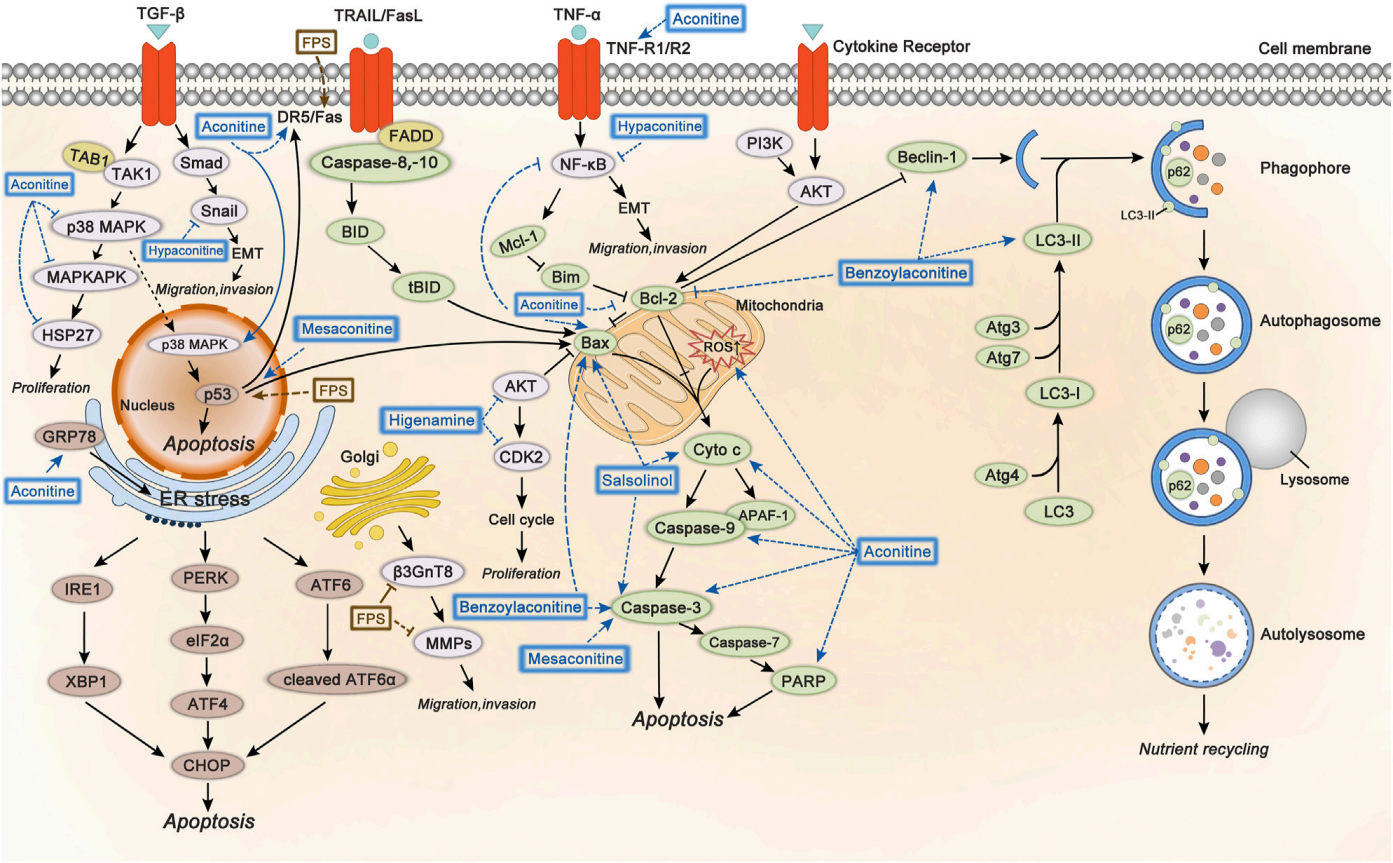
The molecular mechanisms of Fuzi against cancer. 1) Fuzi induced cell autophagy and conducted apoptosis by targeting mitochondrial-mediated pathway and death receptor-mediated pathway. 2) Fuzi inhibited cell proliferation, migration, and invasion via regulating p38 MAPK, AKT, EMT, β3GnT8 and MMPs. The blue and brown solid arrows indicated direct targets of Fuzi alkaloids and FPS respectively. Correspondingly, the blue and brown dotted ones indicated downstream effects induced by Fuzi alkaloids and FPS.

The involved mechanisms of inducing apoptosis and autophagy, inhibiting cell proliferation, migration, and invasion through different molecular pathways by Fuzi alkaloids and FPS are illustrated in Figure 5.

### Regulate Immunity

The immune escape of tumor refers to the phenomenon that tumor cells escape the recognition and attack of immune system through a variety of mechanisms, so as to survive and proliferate in the body. Some cytokines, such as interleukin-2 (IL-2), IL-6, and IL-12, are able to enhance immune response. In contrast, regulatory T cells (Tregs) could rapidly detect and inhibit IL-2 or other cytokines in the early stage of the immune response to suppress autoimmunity, which might hinder the anticancer immunity (Wing et al., 2019). Therefore, inhibiting the production of Tregs is one of the methods of anticancer immunotherapy. Fuzi aqueous extract has been proved to enhance the anticancer effect of radiotherapy in the treatment of lung cancer. The experimental data indicated that ionizing radiation affected the anticancer immune response of mice. However, after the administration of Fuzi aqueous extract, the levels of IL-2, IL-6, and IL-12 in mice serum increased, which might arouse the immune response. Moreover, Fuzi aqueous extract reduced the radiation-induced production of IL-10, TGF-β, and Tregs, revealing the mechanism of Fuzi in modulating immunity and inhibiting tumor growth (Zhang et al., 2017). Another research showed that, in gastric cancer-bearing mice, aconitine was involved in the intervention of Tregs by regulating the prostaglandin E2/cyclooxygenase-2 (PGE2/COX-2) pathway. It was noteworthy that high-dose aconitine reduced PGE2 and Tregs significantly, whose effect was more obvious than that of the celecoxib-positive group. These results suggested that aconitine exerted antitumor efficacy by reversing immune escape through regulating Tregs and mediating the PGE2/COX-2 pathway (Cheng, 2019).

**FIGURE 6:**
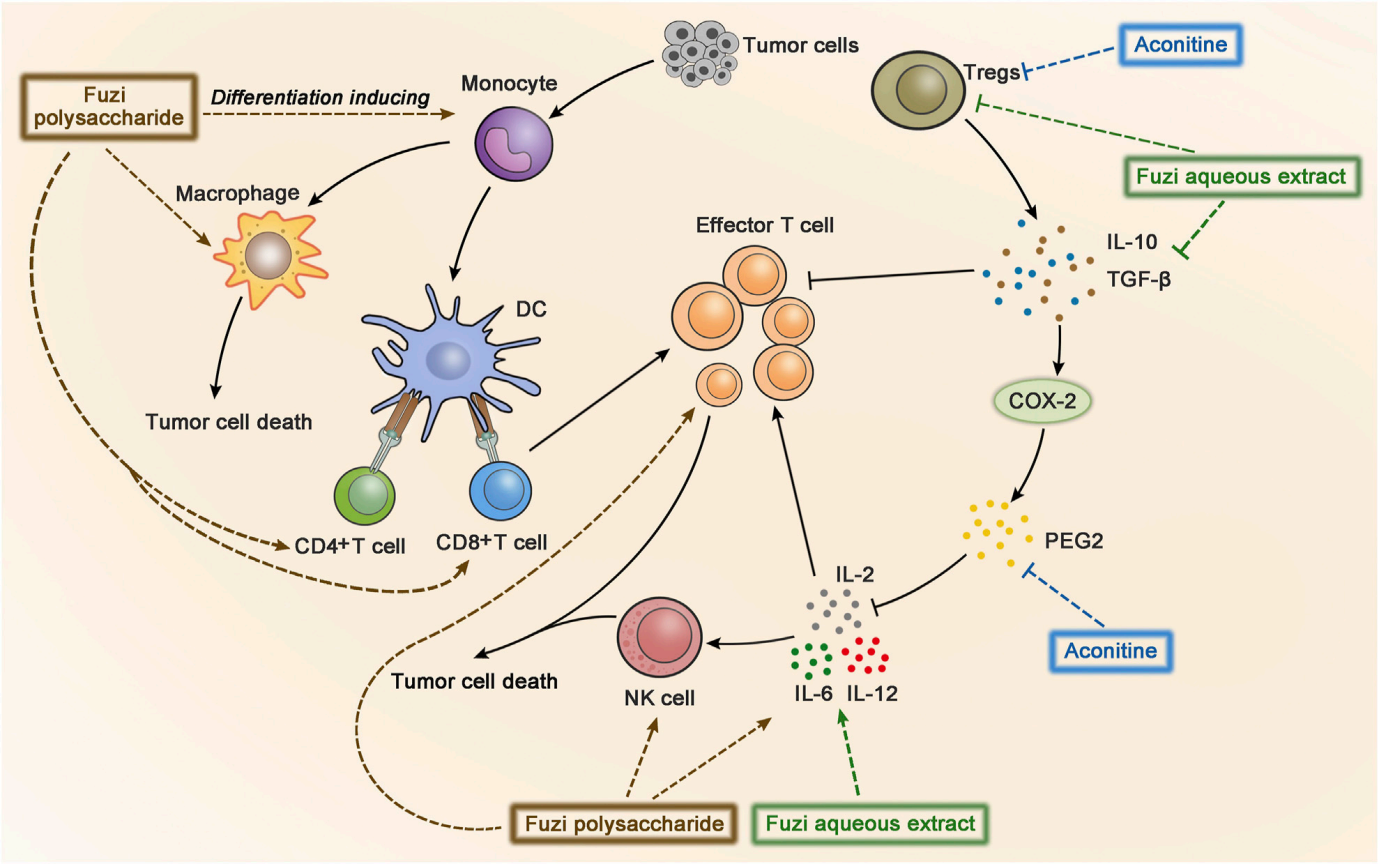
The anticancer immunomodulatory mechanism of Fuzi. Fuzi induced peripheral blood monocytes to differentiate into DC, activated T lymphocytes, and increased the expression of macrophages, CD4^+^, CD8^+^ T cells, NK cells, IL2, IL-12, IL-6, etc. Besides, Fuzi reduced the production of IL-10, TGF-β, Treg, and arrested PGE2/COX-2 pathway. The blue, brown and green dotted arrows indicated the influence induced by alkaloids, FPS and Fuzi aqueous extract respectively.

Cancer immunotherapy aims to promote tumor-specific T-cell response. As antigen-presenting cells, dendritic cells (DC) allow antigens to be recognized by CD4^+^ T cells and CD8^+^ T cells, and then CD4^+^ T cells transmit information to CD8^+^ T cells and facilitate the differentiation of effector T cells to kill tumor cells (Borst et al., 2018). Aconitine possessed a direct anticancer activity, but it also had the drawback of damaging immunity to a certain extent (Qian, 2015). FPS increased the number of macrophages, CD4^+^, and CD8^+^ T cells in the spleen of Hepa1-6 tumor-bearing mice, indicating FPS had immune-improving functions. The results suggested that FPS combined with Fuzi alkaloids might achieve better anticancer efficacy (Qian, 2015). Treatment with FPS induced differentiation of peripheral blood monocytes to DC and expressed its mature phenotypes, which acted as the second signal to activate T lymphocytes and stimulated tumor immunity (Gao L. L. et al., 2012). The synergic effect of FPS and adriamycin was evaluated in H22 tumor-bearing mice. The killing activity of NK cells and lymphocyte transformation rate were dramatically improved; meanwhile the expression of IL-2 and IL-12 in splenic lymphocytes increased, indicating that FPS enhanced the anticancer effect of adriamycin by strengthening the immune system (Dong et al., 2003b; Dong et al., 2006). The anticancer immunomodulatory mechanism of Fuzi is shown in Figure 6.

### Affect Energy Metabolism

The abnormal energy metabolism is one of the major characteristics of tumor cells (Hanahan and Weinberg, 2011). The main sources of biological energy depend on central carbon metabolism, including aerobic respiration and glycolysis. In the presence of adequate oxygen, pyruvate, transformed from glucose, is further oxidatively phosphorylated by mitochondrial respiration. However, under hypoxic conditions, pyruvate would be reduced to lactate. Tumor cells tend to obtain ATP through glycolysis even under aerobic conditions, this phenomenon is called Warburg effect (Warburg et al., 1927). PI3K/AKT/mTOR and HIF-1 pathways would influence glycolysis and participate in the energy metabolism of tumor cells. The PI3K/AKT/mTOR pathway could activate glucose transporters on cell membrane and the metabolic enzyme hexokinase 2 (HK2) in glycolysis, sequentially increase glucose uptake and glycolysis rate (Gottlob et al., 2001; Roberts et al., 2013; Abdel-Wahab et al., 2019). The HIF-1α could induce the activation of lactate dehydrogenase A (LDHA) and pyruvate dehydrogenase kinase 1 (PDK1) to catalyze the conversion of pyruvate to lactate, accordingly promote glycolysis to produce ATP in the anoxic tumor microenvironment (Valvona et al., 2016; Zhou et al., 2018; Nagao et al., 2019).

**FIGURE 7:**
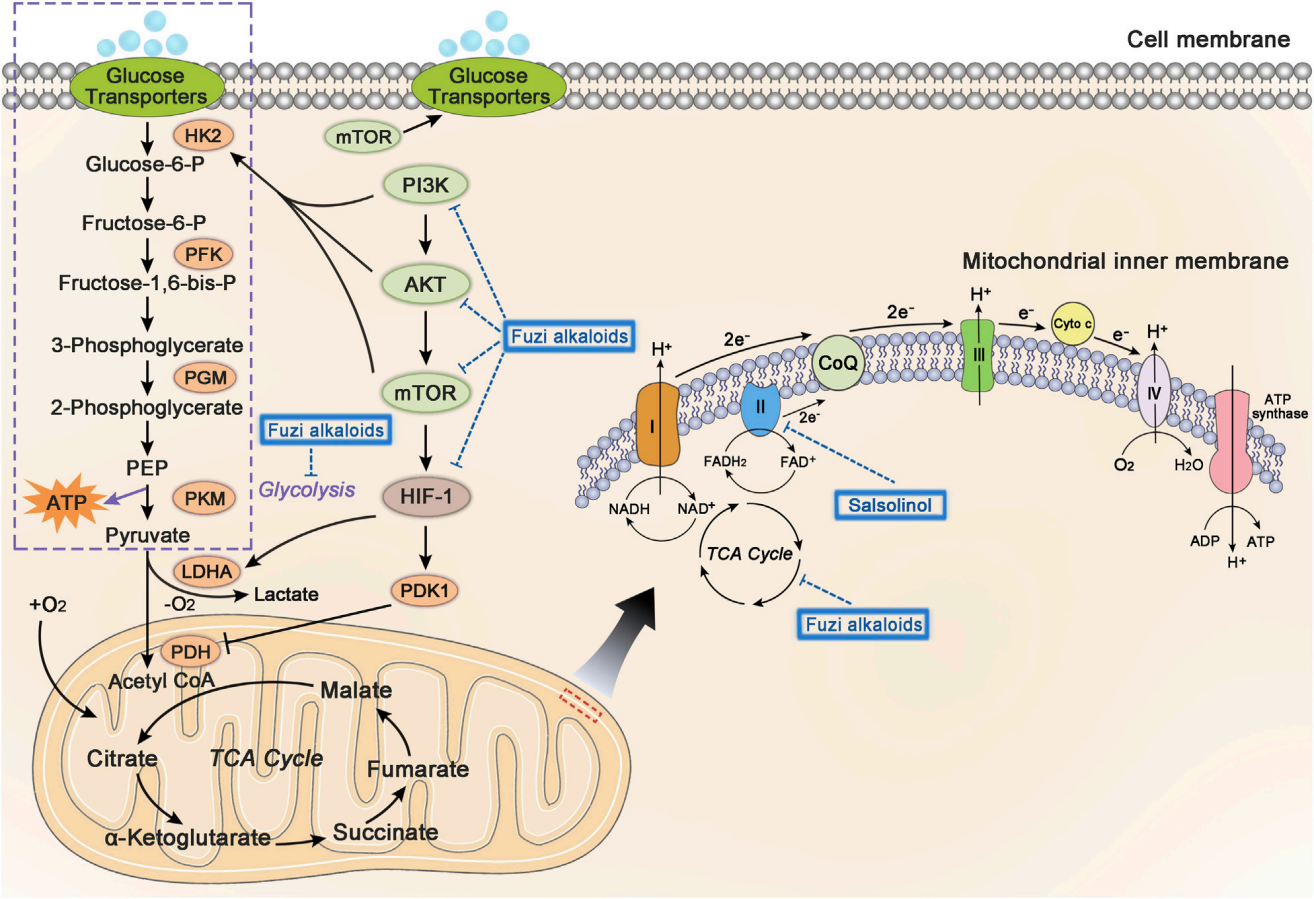
The influence of Fuzi alkaloids on cancer energy metabolism. Fuzi regulated the energy supply of cancer cells, as well as induced cell death by inhibiting mitochondrial complex II, PI3K/AKT/mTOR pathway, HIF-1 pathway, and central carbon metabolism. The blue dotted arrow indicated the effect induced by Fuzi alkaloids.

Mitochondria are the most important organelle of energy metabolism. Besides supplying energy for life, mitochondria also regulate cell death, control redox reactions, and provide substrates for anabolism (Porporato et al., 2018). Angiogenesis in the center of many solid tumors is poor, resulting in limited supply of glucose and oxygen. However, electron transport chain (ETC) in the mitochondria could function even under hypoxia, allowing the tumor cells to breathe and produce ATP (Vasan et al., 2020). Complex I, II, III, and IV, located on the mitochondrial inner membrane, are key components of ETC to transfer electrons. Complex I and II respectively oxidize the NADH and FADH_2_ to NAD^+^ (nicotinamide adenine dinucleotide) and FAD^+^ (flavin adenine dinucleotide), which keep TCA cycle running. Complex I, II, III, and IV transfer electrons to oxygen, discharge protons into the gap of mitochondrial membrane, and eventually generate ATP by ATP synthase (Letts and Sazanov, 2017). Regulation of cellular metabolism might be a significant clue in developing antineoplastic agents (Stuart et al., 2014; Wheaton et al., 2014; Kuntz et al., 2017). Figure 7 shows glycolysis, TCA cycle, and relevant pathways involved in cellular energy metabolism.

Existing studies found that Fuzi alkaloids regulated cellular metabolism by affecting ETC in the mitochondria. Salsolinol blocked the energy supply of SH-SY5Y cells and induced cell death. It was noteworthy that adding d-glucose to enhance glycolysis could not prevent the cytotoxicity of salsolinol on SH-SY5Y cells. Further research demonstrated that salsolinol inhibited the activity of complex II (succinate-Q reductase) and caused a rapid depletion in intracellular ATP, which might account for the cytotoxic effects of salsolinol (Storch et al., 2000). Moreover, network pharmacology predicted that Fuzi alkaloids might exert antitumor effects by suppressing the PI3K/AKT/mTOR pathway, HIF-1 pathway, and central carbon metabolism in cancer (Lu et al., 2021) (Figure 7). The evidence proved that energy metabolism and its relevant signal pathways might be involved in the anticancer process of Fuzi alkaloids.

### Reverse Multidrug Resistance

Multidrug resistance (MDR) is a principal reason of the therapeutic failure. P-glycoprotein (P-gp) and breast cancer resistance protein (BCRP) located on the cell membrane control the absorption, distribution, and excretion of various chemicals. These transporters protect cancer cells from high doses of intracellular drugs and lead to the occurrence of MDR (Bukowski et al., 2020). Many inhibitors, such as verapamil and cyclosporine A, are designed to target P-gp. However, due to the low affinity of these inhibitors to P-gp, high concentrations are required and side effects would ensue (Höllt et al., 1992; List et al., 2001; Waghray and Zhang, 2018). Aconitine was shown to reverse the MDR of KB_V200_ cells, accordingly the inhibitory rate by aconitine reached 56.15% at 50 μg/ml. The combined use of aconitine and vincristine achieved a better reversal effect of MDR, largely due to downregulating P-gp expression (Liu et al., 2004).

The anticancer active compounds of Fuzi and their mechanisms are summarized in Table 4.

**TABLE 4:** Anticancer constituents of Fuzi and relevant mechanisms.

Constituent	Cells or cancer models	Molecular mechanisms	Reference
***Induce apoptosis and autophagy***			
Aconitine	HepG2 cells	Upregulated the expression of cleaved PARP, caspase-3, caspase-7, and Bax and downregulated the expression of Bcl-2	Qi et al. (2018)
Salsolinol	SH-SY5Y cells	Upregulated the expression of Cyto C and Bax and downregulated the expression of Bcl-2	Wanpen et al. (2007)
Salsolinol	SH-SY5Y cells	Increased the release of caspase-3	Jantas et al. (2008)
Aconitine	A549 cells	Increased the expression of p38 MAPK, DR5, and TNF-R1	Fan et al. (2016)
Mesaconitine	K562, K562 daunorubicin-resistant cells	Increased the expression of caspase-3 and p53 to trigger death receptor-mediated apoptosis	Guan et al. (2017)
FPS	S180, H22 tumor-bearing mice	Increased the expression of p53 and Fas	Dong et al. (2003a)
Aconitine	HeLa cells	Increased the expression of eIF2α, ATF4, IRE1, XBP1, ATF6, PERK, and CHOP	Li et al. (2018)
Benzoylaconitine	A549 cells	Upregulated the expression of Beclin1, LC3-Ⅱ, Bax, and caspase-3 and downregulated the expression of p62 and Bcl-2	Shao et al. (2019)
Aconitine	Miacapa-2, PANC-1 cells	Upregulated the expression of Bax, caspase-9, caspase-3, PARP, and Cyto C and downregulated the expression of Bcl-2 and NF- κB	Ji et al. (2016)
***Inhibit proliferation, migration, and invasion***			
Aconitine	MHCC97 cells	Inhibited the P38 MAPK pathway by suppressing the phosphorylation of p38, MAPKAPK, and HSP27	Xiong et al. (2018)
Higenamine	SKBr3, T47D cells	Decreased the expression of p-AKT and p-CDK2	Jin et al. (2018)
Hypaconitine	A549 cells	Suppressed EMT by reducing the expression of Snail and NF-κB	Feng et al. (2017)
FPS	SK-HEP-1 cells	Downregulated the expression of β3GnT8 and polylactosamine	Gao et al. (2016)
FPS	Gastric cancer xenografts in nude mice	Suppressed the expression of MMP-2 and MMP-14	An et al. (2019)
***Regulate immunity***			
Fuzi aqueous extract	Lewis cells	Increased the release of IL-2, IL-5, IL-6, and IL-12 and decreased the release of IL-10, TGF-β, and Tregs	Zhang et al. (2017)
Aconitine	MFC tumor-bearing mice	Downregulated the expression of PGE2 and Tregs	Cheng (2019)
FPS	Hepa1-6 tumor-bearing mice	Increased the number of macrophages and CD4^+^ and CD8^+^ T cells in spleen	Qian (2015)
FPS	Peripheral blood monocytes	Induced differentiation of peripheral blood monocytes to DC	Gao et al. (2012b)
FPS	S-180, H22 tumor-bearing mice	Increased the killing activity of NK cells, the transformation rate of T cells, and the expression of IL-2 and IL-12	Dong et al. (2003b); Dong et al. (2006)
***Affect energy metabolism***			
Salsolinol	SH-SY5Y cells	Suppressed the activity of succinate-Q reductase	Storch et al. (2000)
***Reverse multidrug resistance***			
Aconitine	KB_V200_ cells	Downregulated the expression of P-gp	Liu et al. (2004)

## Conclusion

Natural plants have attracted significant interest ascribed to their anticancer properties. In this review, we rounded up the available evidence on the antineoplastic efficacies of Fuzi. As mentioned above, Fuzi alkaloids and polysaccharides showed tumor-suppressive effects *in vitro* and *in vivo* by inducing apoptosis and autophagy, inhibiting cell proliferation, migration and invasion, regulating immunity, affecting energy metabolism, and reversing MDR. Several signaling pathways and biological processes were involved in these pharmacological functions, such as NF-κB, EMT, HIF-1, p38 MAPK, PI3K/AKT/mTOR, and TCA cycle. The anticancer molecular mechanism of Fuzi alkaloids and polysaccharides concluded in this review could lay a foundation for further basic research and clinical application.

Outstanding achievements have been obtained in the anticancer chemical elements and pharmacological mechanism of Fuzi. Nevertheless, there are some deficiencies in current research. Nowadays, researchers focus on alkaloid monomers, crude polysaccharide or Fuzi extract. Other constituents such as flavonoids, ceramides and fatty acids are seldomly investigated about their anticancer activity. According to the characteristics of multi-components and multi-targets of TCM, the combination efficacy and network regulation mechanism of bioactive constituents in Fuzi remain to be further studied. With the applications of diversified techniques, specific mechanisms such as drug ligand-receptor interactions and conformational changes of targets are expected to be clarified. To sum up, Fuzi and its active ingredients would serve as attractive therapeutic candidates for the development of anticancer drugs.
